# Effects of roadside habitat management on epigeic arthropod diversity: a case study from the Nitra-Selenec expressway junction

**DOI:** 10.1038/s41598-026-54425-z

**Published:** 2026-06-22

**Authors:** Juraj Litavský, Oto Majzlan, Vladimír Langraf, Samuel Iracký, Božena Šerá

**Affiliations:** 1 Department of Environmental Ecology and Landscape Management, Faculty of Natural Sciences, Comenius University in Bratislava, Mlynská dolina, Ilkovičova 6, Bratislava, 842 15 Slovakia; 2 Faculty of Natural Sciences and Informatics, Constantine the Philosopher University in Nitra, Trieda Andreja Hlinku 1, Nitra, 949 74 Slovakia

**Keywords:** Road verges, Habitat management, Mulching, Biodiversity, Opiliones, Carabidae, Ecology, Ecology, Zoology

## Abstract

Linear transport infrastructure fragments habitats, but its edges can serve as significant refuges for invertebrates. Management of these verges is crucial to realise this conservation potential, but the impact of specific habitat measures on epigeic arthropods remains poorly understood. This study assessed the impact of roadside habitat management on the diversity and composition of epigeic arthropods, using ground beetles (Carabidae) and harvestmen (Opiliones) as bioindicators at the Nitra-Selenec expressway junction, Slovakia. Over two years, we used pitfall traps to sample epigeic arthropods at ten sites managed under three different regimes: passive management (no intervention), active management with renewal/seeding (commercial grass-herb mixture), and active management with mulching only. We analysed the influence of management, vegetation structure, and landscape variables on species assemblages using redundancy analysis and predicted population trends using machine learning. We recorded 1,416 carabids (50 species) and 1,409 harvestmen (6 species). The renewal/seeding intervention had a significant negative effect on the community composition. The structure of the vegetation, specifically the cover of the herb layer and species richness of the shrub layer, were the most significant positive drivers of community assembly. Furthermore, distance from the road significantly influenced species distribution. Analysis of population trends revealed a gradual increase in carabid abundance over time, but an alarming decline in harvestmen. Active revegetation with commercial seed mixtures creates a homogeneous habitat that is less suitable for diverse epigeic communities than passive management. The structural complexity provided by various native vegetation is a key factor in supporting invertebrates. Implications for insect conservation: We recommend that roadside managers prioritise passive management or regionally appropriate native seed mixtures over commercial revegetation, maintain structural complexity of vegetation through a reduced frequency of mowing (1–2 times annually at ≥ 10 cm height), and adopt mosaic approaches that combine intensive mowing only in safety-critical zones with extensive management elsewhere.

## Introduction

The effects of roads on biodiversity have become a central topic in ecological research. As pervasive landscape elements, roads act as potent agents of habitat fragmentation, physical barriers, and sources of environmental stressors such as pollution and noise, collectively impacting the diversity and abundance of invertebrates communities^1–3^; Anderson et al., 2017^4^;. These impacts often lead to increased mortality, disrupted movement, and altered microhabitats, contributing to declines in species richness and population stability^5^; Skórka^6^; Anderson^7^).

Paradoxically, the infrastructure that fragments habitats also creates new habitats. Road verges account for up to 0.2% of the global land area, representing a substantial surface when viewed globally and consequently attracting attention for their potential to support biodiversity (Noordijk et al.^8–11^^12^,). The role of road verges as potential habitats and dispersal corridors for ground beetles has been recognised for decades. Pioneering work by Vermeulen^13^^14^, demonstrated that roadside verges can function as corridors for stenotopic heathland carabids, facilitating movement between isolated habitat patches. Modelling approaches have further confirmed that road verges can support dispersal of carabid beetles, with movement patterns influenced by verge structure and habitat quality^15^. Subsequent research confirmed that the corridor function depends on verge structure, connectivity, and the dispersal ability of individual species^16,17^. These roadside habitats, including verges, embankments, and drainage systems, are subject to frequent disturbances from road maintenance, pollution, and vehicular traffic. Despite these challenges, they can serve as critical refuges for invertebrates, including pollinators, decomposers, and predators, which play essential roles in ecosystem function^18–20^. More recent studies have examined the impacts of road construction and modernisation on carabid assemblages, showing that such interventions can significantly alter species composition depending on the structure of the surrounding landscape^21^. However, the ecological value of these habitats is highly dependent on management practices, which can either enhance or diminish their capacity to sustain diverse invertebrate communities^22,23^. Therefore, environmental monitoring and assessment of these practices are essential for evidence-based roadside management.

Invertebrates, providing indispensable ecosystem services from nutrient cycling to pest control, are vital to ecosystem stability^24–26^. Their ability to respond to environmental changes makes them valuable bioindicators for assessing habitat quality and ecosystem integrity^27,28^. Among them, ground beetles (Carabidae) and harvestmen (Opiliones) are particularly well-suited as bioindicators for evaluating disturbed landscapes, such as road edges. Both groups are predominantly predatory or scavengers and are sensitive to environmental alterations, reflecting broader ecosystem conditions^29–37^. They share similar trophic roles, feeding primarily on small soft-bodied invertebrates, although many species also act as opportunistic scavengers or consume plant matter^38–42^. However, a key ecological difference lies in their dispersal capacity: most carabid beetles are capable of flight, facilitating the colonisation of isolated patches, whereas harvestmen are wingless and less mobile. This makes harvestmen more vulnerable to habitat fragmentation and makes them potent indicators of local habitat quality and landscape connectivity^30,32,35,36,43^.

Despite the recognised importance of roadside management, there is a critical lack of specific evidence on how common interventions, such as active revegetation with commercial seed mixtures versus passive natural succession, affect these different epigeic arthropod groups. Furthermore, the relative importance of management compared to other factors, such as vegetation structure and landscape context, remains poorly understood. Addressing this knowledge gap requires the systematic monitoring and assessment of arthropod communities under different management regimes.

Therefore, the main objectives of this study were: (1) to compare the composition, richness, and abundance of epigeic harvestmen and ground beetles in different roadside management regimes (passive, active with renewal/seeding, and active with mulching only) at the Nitra-Selenec expressway junction in Slovakia; (2) to identify the key environmental drivers, including management practices, vegetation structure, and landscape variables, shaping these arthropod communities using multivariate analysis; and (3) to analyse the temporal population trends of both taxa to assess the long-term impact of established habitat conditions.

## Materials and methods

### Study area

**Fig. 1:**
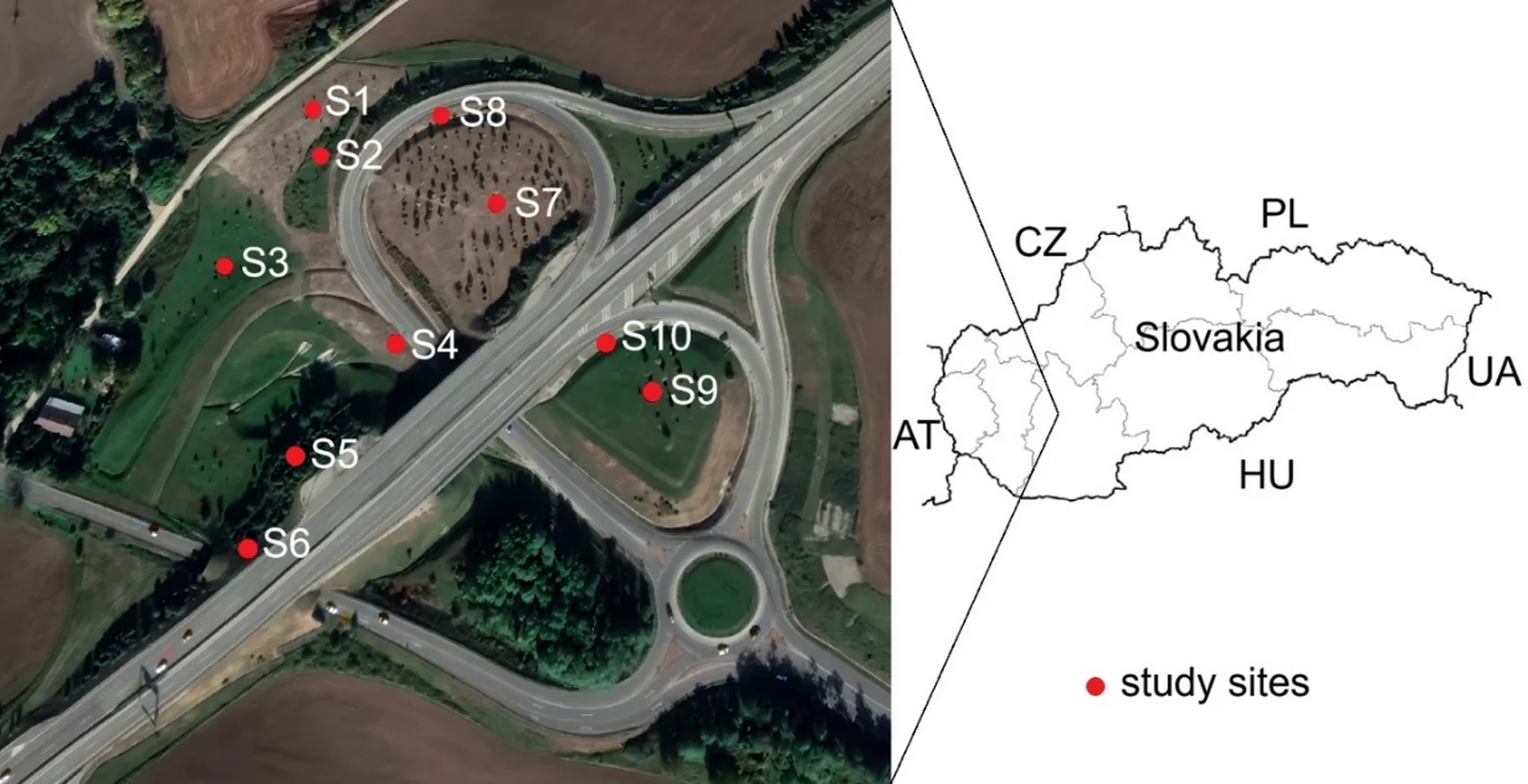
Illustration of the study area within the Nitra-Selenec expressway junction (Slovakia): S1–S10 – study sites, AT – Austria, CZ – Czech Republic, HU – Hungary, PL – Poland, UA – Ukraine.

Research on soil-dwelling harvestmen (Opiliones) and ground beetles (Carabidae) was conducted in selected habitats at the junction of the Nitra-Selenec R expresswayR1 eExpressway, located 1 km east of the city of Nitra (Slovakia). The R1 Expressway is a 45.9 km long dual carriageway that connects Nitra and Tekovské Nemce, located east of Bratislava, the capital city. It was completed in 2011. Within the study area, 10 study sites were established (four in completely isolated vegetation islands and six in open spaces communicating with the surrounding landscape; Fig. 1).

All study sites were arranged as linear transects located away from trees, indicating that the tree layer was completely absent from these areas. However, young trees, not taller than 3 m, could be found in the nearby surroundings.

S1 (48°18’36.91” N, 18°08’23.33” E, 153 m a.s.l.) – this site underwent renewal/seeding and extensive mulching in 2022 and 2023. It was not completely isolated by road construction and was located closest to agricultural land. In the herbaceous layer, 24 plant species were recorded in 2022, which increased to 32 in 2023. The dominant species included *Elytrigia repens*, *Stellaria media*, and *Taraxacum* sect. *Taraxacum*. The shrub layer was absent at this site.

S2 (48°18’35.84” N, 18° 8’23.49” E, 154 m a.s.l.) – a non-isolated study site representing young shrubs planted in 2018 with passive management. The shrub layer consisted of *Crataegus* sp., *Forsythia* sp., *Ligustrum vulgare*, and *Spiraea* sp., whereas the herbaceous layer was dominated by *Festuca rupicola* and *Trifolium pratense*.

S3 (48°18’33.67” N, 18°8’21.45” E, 153 m a.s.l.) – an unisolated site where no renewal/seeding was performed. It was located closest to a forest fragment and farthest from the nearest road (asphalt or concrete). This area was intensively mulched with 100% plant coverage in the herbaceous layer. The dominant species in the herbaceous layer included *Trifolium pratense*, *Medicago lupulina*, *Poa pratensis*, *Plantago lanceolata*, *Taraxacum* sect. *Taraxacum*, and *Trifolium repens*.

S4 (48°18’32.45” N, 18°8’26.02” E, 159 m a.s.l.) - an unisolated study site where renewal/seeding was conducted. The site underwent extensive mulching in 2022 and intensive mulching in 2023. It was the farthest from agricultural land among the study sites.

In the herbaceous layer, 28 plant species were recorded in 2022 and 29 species in 2023. The dominant species were *Cerastium brachypetalum*, *Lactuca saligna*, *Veronica arvensis*, and *Veronica persica*.

S5 (48°18’30.73” N, 18° 8’23.66” E, 157 m a.s.l.) – an unisolated study site where shrubs were planted in 2012/2013, and no further management (no mulching) was carried out. The shrub layer consists of species such as *Fontanesia fortunei*, *Ligustrum vulgare*, and *Prunus spinosa*, with coverages of 60% in 2022 and 70% in 2023.

S6 (48°18’29.33” N, 18°8’22.83” E, 158 m a.s.l.) – an unisolated study site located 3.1 m from the expressway. No renewal/seeding was performed at this site. The area was extensively mulched. The coverage of the herb layer reached 80%, with the following plant taxa being the most represented in this layer: *Festuca rubra* agg., *Bromus hordeaceus*, *Poa pratensis*, and *Achillea millefolium* agg.

S7 (48°18’34.96"N, 18°08’27.97"E, 160 m a.s.l.) - an isolated study site where renewal/seeding was implemented. The site was extensively mulched during both years of the study. Here was recorded the highest plant species richness in the herb layer (37 species in 2022 and 36 species in 2023), dominated by *Cerastium brachypetalum*, *Noccaea perfoliata*, *Veronica arvensis*, and *Veronica persica*.

S8 (48°18’36.81"N, 18°08’26.93"E, 156 m a.s.l.) - an extensively mulched, isolated site where no renewal/seeding was implemented. The site was located at the edge of the road. The herb layer was dominated by *Helminthotheca echioides*. The shrub layer was absent at this site.

S9 (48°18’31.45"S, 18°08’31.62"E, 169 m a.s.l.)–an intensively mulched, isolated site where no restoration/seeding was conducted. This site was the farthest from the nearest forest fragment. The herb layer achieved 90% coverage, with *Trifolium pratense* and *Leontodon hispidus* being the most dominant species. The shrub layer was formed by *Acer pseudoplatanus*.

S10 (48°18’32.38"N, 18°08’30.49"E, 167 m a.s.l.) - an isolated, intensively mulched site where no renewal/seeding was conducted. The site was located closest to the road edge (expressway). The lowest plant species richness (16 species) was recorded at this study site. *Elytrigia repens* dominated in the herb layer. A shrub layer was absent.

### Methods

Entomological research was conducted from March 24, 2022, to January 17, 2024. Epigeic harvestmen (Opiliones) and ground beetles (Carabidae) were captured using pitfall traps^44^, consisting of half-litre plastic cups with an inner diameter of 9 cm, which were filled halfway with a 4% formaldehyde solution. The solution was supplemented with antifreeze (FRIDEX) and a few drops of detergent. Each trap was equipped with a cover to prevent the dilution of the solution by atmospheric precipitation. Trap contents were collected at regular monthly intervals. Five traps were placed in a line on each study plot, with a minimum distance of 5 m between them. The contents of the traps were merged for each site and date to form a unified sample for each collection period. The samples were sorted in the laboratory and then identified to the species level. Ground beetles were identified according to Hůrka^45^, and Müller-Motzfeld^46^, and harvestmen according to Martens^47^ and Litavský^48^. The samples were preserved in 75% ethanol. A list of ground beetle species was arranged according to Lorenz^49^,and harvestmen according to Kury et al.^50^.

The study sites differed from each other mainly in terms of distance from the nearest road, distance from agricultural land, distance from a forest fragment, cover of herb (E_1_) and shrub (E_2_) layers, richness of plant species in individual layers (E_1_ and E_2_), and type of management (Table 1).

**Table 1:** Selected parameters assessed within each study site (S1–S10): coverage of herbaceous (E_1_ [%]) and shrub (E_2_ [%]) layers and species richness of plants within the herbaceous (E_1_ [spec.]) and shrub (E_2_ [spec.]) layers during 2022 and 2023. Isolation indicates whether the site is completely isolated by road infrastructure (yes = isolated vegetation island; no = open communication with surrounding landscape).

Study sites	S1	S2	S3	S4	S5	S6	S7	S8	S9	S10
renewal/seeding	yes	no	no	yes	no	no	yes	no	no	no
mulching 2022	extensive	none	intensive	extensive	none	extensive	extensive	extensive	intensive	intensive
mulching 2023	extensive	none	intensive	intensive	none	extensive	extensive	extensive	intensive	intensive
isolation	no	no	no	no	no	no	yes	yes	yes	yes
distance from agricultural land [m]	32.6	57.1	87.5	145.7	93.3	56.1	86.1	40.4	92.8	103.9
distance from forest fragment [m]	98.6	96.3	50.6	152.7	130.4	133.2	181.1	172.6	253.3	240.4
distance from the nearest road [m]	35.2	16.3	60.2	2.8	23.2	3.1	51	2.4	40.5	2
E_1_ [spec.] 2022	24	21	21	28	25	19	37	31	19	16
E_2_ [spec.] 2022	0	4	2	0	3	0	1	0	1	0
E_1_ [%] 2022	85	70	100	80	70	80	70	70	90	55
E_2_ [%] 2022	0	60	6	0	60	0	1	0	1	0
E_1_ [spec.] 2023	32	21	21	29	25	19	36	31	19	16
E_2_ [spec.] 2023	0	4	2	0	3	0	1	0	1	0
E_1_ [%] 2023	95	70	100	80	70	80	95	70	90	55
E_2_ [%] 2023	0	75	6	0	70	0	1	0	1	0

**Table:** 

Study sites	S1	S2	S3	S4	S5	S6	S7	S8	S9	S10
renewal/seeding	yes	no	no	yes	no	no	yes	no	no	no
mulching 2022	extensive	none	intensive	extensive	none	extensive	extensive	extensive	intensive	intensive
mulching 2023	extensive	none	intensive	intensive	none	extensive	extensive	extensive	intensive	intensive
isolation	no	no	no	no	no	no	yes	yes	yes	yes
distance from agricultural land [m]	32.6	57.1	87.5	145.7	93.3	56.1	86.1	40.4	92.8	103.9
distance from forest fragment [m]	98.6	96.3	50.6	152.7	130.4	133.2	181.1	172.6	253.3	240.4
distance from the nearest road [m]	35.2	16.3	60.2	2.8	23.2	3.1	51	2.4	40.5	2
E_1_ [spec.] 2022	24	21	21	28	25	19	37	31	19	16
E_2_ [spec.] 2022	0	4	2	0	3	0	1	0	1	0
E_1_ [%] 2022	85	70	100	80	70	80	70	70	90	55
E_2_ [%] 2022	0	60	6	0	60	0	1	0	1	0
E_1_ [spec.] 2023	32	21	21	29	25	19	36	31	19	16
E_2_ [spec.] 2023	0	4	2	0	3	0	1	0	1	0
E_1_ [%] 2023	95	70	100	80	70	80	95	70	90	55
E_2_ [%] 2023	0	75	6	0	70	0	1	0	1	0

The study sites were divided into three categories based on the implemented management measures.

Sites with passive management – sites with planted shrubs without mulching of the undergrowth.

Sites with active management were categorised into two types:

- Sites with renewal/seeding: These sites were seeded with a mixture of 41 higher plant taxa, comprising 90% grasses and 10% herbs, of which 2% were legumes. The sites were extensively or intensively mulched. Extensive mulching involved mulching up to twice a year with a cutting height of at least 10 cm above the soil surface, whereas intensive mulching involved three or more mulching events per year at a lower height above the soil surface.
- Sites without seed mixture sowing: These sites received extensive or intensive mulching but did not have a seed mixture applied.

The renewal/seeding management measure was implemented within the Nitra-Selenec expressway junction at three sites with a total area of 16,000 m². The process involved the following steps: in autumn 2021, the sites were mowed, followed by the application of a non-selective herbicide. Two weeks later, the terrain was adjusted, the soil was cultivated with a cultivator, and a grass-herb mixture for dry lawns “Paprsek” (from fa AGROSTIS) was seeded at a rate of 8–10 g/m². The sites were then rolled up. Three study sites (S1, S4, and S7) were established in these managed areas.

Temperature data were recorded every 15 min during the entomological investigation using TMS-4 data loggers^51^ placed at the centre of each monitored transect. The TMS-4 data loggers measured soil temperature at a depth of 6 cm, air temperature at ground level + 2 cm, and 15 cm above ground. For our analysis, we considered only the air temperature recorded 15 cm above the soil surface. Furthermore, soil moisture was measured at a depth of 11 cm, but the recorded soil moisture values were not evaluated because some moisture sensors did not record moisture at certain times. Therefore, we aimed to avoid incorrect data interpretation.

According to Hůrka^45^, Matalin^52^, Müller-Motzfeld^46^, Holecová^42^,Talarico et al,)^53^, and our own observations, we classified the ground beetle (Carabidae) species in Table 2 according to their feeding habits as zoophagous (z), phytophagous (ph), and omnivorous (om). Carabid species were classified according to their dispersal ability based on wing morphology, following established criteria in the literature^42,45,46,52,54^, supported by our own field observations. Three categories were distinguished: F (flying) – macropterous species with fully developed wings capable of active flight; N (non-flying) – apterous or brachypterous species that have reduced second pairs of wings and are functionally flightless; B (brachypterous, occasionally able to fly) – species that are predominantly brachypterous and generally considered flightless^54^, but exhibit intraspecific variability, with rare individuals possessing fully developed (macropterous) wings capable of flight^45,52^. We also added information to this table on the feeding habits of harvestmen (Opiliones) based on Pinto-da-Rocha et al.^35^, and our own observations.

### Statistical analyses

The collected data were analysed using multivariate analysis, in which we sought dependencies between objects (species of the Carabidae family and the Opiliones order) and environmental variables (renewal/seeding, mulching in 2022 and 2023, isolation, distance from agricultural land, distance from forest fragment, distance from the nearest road, plant diversity, and cover of the herbaceous and shrub vegetation layers). Based on the determined length of the gradient (SD = 2.2 on the first ordination axis), we used a linear method to evaluate the materials. The statistical significance of the variables was tested using the Monte Carlo permutation test (499 iterations) in the Canoco5 programme^55^. For data evaluation, we used: Redundancy Analysis (RDA - Redundancy Analysis). We also performed statistical data analysis using Python 3.12^56^. The analysis focused on multivariable linear regression. To analyse the development of the number of individuals in time series, the K-means clustering method was applied, enabling the identification of groups of time series with similar developmental patterns. The optimal number of clusters (k) was determined using the Elbow Method, based on the analysis of the relationship between the number of clusters and the within-cluster sum of squares (WCSS). The final number of clusters was selected by identifying the point of inflection (“elbow”), where the decrease in WCSS begins to slow down^57^.

## Results

At the ten study sites, we recorded 1,416 individuals of the Carabidae family belonging to 50 species (Table 2). The eudominant species were *Harpalus distinguendus* (16.82%), and *Harpalus rufipes* (13.99%). From the order Opiliones, we recorded 1,409 individuals belonging to six species. The eudominant species was *Opilio saxatilis* (86.95%).

**Table 2:** A list of the species of harvestmen and carabids obtained at the study sites (S1–S10) in the years 2022–2024 in the Nitra-Selenec expressway junction, supplemented with their feeding habits and flying ability. FH feeding habits: zoophagous (z), phytophagous (ph), omnivorous (om); FL flying ability: flying (F), flightless (N), brachypterous, occasionally capable of flying (B).

Taxa	Abbr.	FH	FL	S1	S2	S3	S4	S5	S6	S7	S8	S9	S10	Individuals ∑	%
Carabidae															
*Abax parallelepipedus* (Piller & Mitterpacher, 1783)	AbaxParl	z	N	1	0	2	0	0	0	0	0	0	0	3	0.21%
*Acupalpus flavicollis* (Sturm, 1825)	AcupFlav	om	F	6	0	0	0	0	2	0	0	0	0	8	0.57%
*Acupalpus maculatus* (Schaum, 1860)	AcupMacl	om	F	0	0	0	0	1	0	0	1	0	0	2	0.14%
*Amara aenea* (DeGeer, 1774)	AmarAene	ph	F	7	8	3	0	0	0	13	0	1	0	32	2.26%
*Amara aulica* (Panzer, 1796)	AmarAulc	ph	F	4	0	4	0	0	0	1	0	0	0	9	0.64%
*Amara familiaris* (Duftschmid, 1812)	AmarFaml	ph	F	0	0	0	0	0	0	0	0	1	0	1	0.07%
*Amara similata* (Gyllenhal, 1810)	AmarSiml	ph	F	0	0	2	0	0	0	0	0	0	0	2	0.14%
*Anchomenus dorsalis* (Pontoppidan, 1763)	AnchDors	z	F	6	10	4	7	0	1	0	0	0	0	28	1.98%
*Aptinus bombarda* (Illiger, 1800)	AptnBomb	z	N	0	1	0	0	0	0	0	0	0	0	1	0.07%
*Badister bullatus* (Schrank, 1798)	BadsBull	z	F	1	0	3	0	1	0	0	0	0	0	5	0.35%
*Bembidion obtusum* Audinet-Serville, 1821	BembObts	z	B	0	0	0	0	1	0	0	0	0	0	1	0.07%
*Bembidion varium* (Olivier, 1795)	BembVari	z	F	1	0	0	2	0	0	0	0	0	0	3	0.21%
*Bradycellus caucasicus* (Chaudoir, 1846)	BradCauc	om	B	1	3	0	2	0	0	0	0	0	0	6	0.42%
*Bradycellus ruficollis* (Stephens, 1828)	BradRufc	om	F	0	0	0	2	0	0	0	4	0	0	6	0.42%
*Brachinus crepitans* (Linnaeus, 1758)	BracCrep	z	F	4	2	0	8	1	13	0	1	0	0	29	2.05%
*Brachinus elegans* Chaudoir, 1842	BracEleg	z	F	2	3	1	0	0	1	1	0	0	0	8	0.57%
*Brachinus explodens* Duftschmid, 1812	BracExpl	z	F	5	4	5	0	0	0	1	1	0	0	16	1.13%
*Calathus erratus* (C.R.Sahlberg, 1827)	CallErrt	z	B	23	16	6	0	0	0	24	2	12	0	83	5.86%
*Calathus fuscipes* (Goeze, 1777)	CaltFusc	z	B	4	0	57	1	0	0	19	0	7	0	88	6.22%
*Calathus melanocephalus* (Linnaeus, 1758)	CaltMeln	z	B	0	0	0	0	0	0	1	1	0	0	2	0.14%
*Carabus scheidleri* Panzer, 1799	CarbSche	z	N	2	1	2	0	0	0	0	1	0	0	6	0.42%
*Carabus violaceus* Linnaeus, 1758	CarbViol	z	N	9	8	1	0	3	6	7	0	0	0	34	2.40%
*Cylindera germanica* (Linnaeus, 1758)	CylnGerm	z	F	3	2	1	2	0	0	0	0	0	0	8	0.57%
*Demetrias atricapillus* (Linnaeus, 1758)	DemtAtrc	z	F	0	4	0	0	0	0	0	0	0	0	4	0.28%
*Harpalus affinis* (Schrank, 1781)	HarpAffn	om	F	17	9	1	0	0	0	0	3	0	12	42	2.97%
*Harpalus distinguendus* (Duftschmid, 1812)	HarpDist	om	F	147	7	10	10	1	3	38	2	12	8	238	16.82%
*Harpalus hirtipes* (Panzer, 1796)	HarpHirt	om	F	0	0	4	1	1	0	1	0	0	1	8	0.57%
*Harpalus rubripes* (Duftschmid, 1812)	HarpRubr	om	F	18	19	19	2	0	3	26	12	26	5	130	9.19%
*Harpalus rufipes* (DeGeer, 1774)	HarpRufp	om	F	83	55	4	4	2	12	35	2	1	0	198	13.99%
*Harpalus tardus* (Panzer, 1796)	HarpTard	om	F	15	9	16	7	2	1	1	2	0	4	57	4.03%
*Chlaenius nigricornis* (Fabricius, 1787)	ChlaNigr	z	F	1	0	0	0	0	0	0	0	0	0	1	0.07%
*Leistus ferrugineus* (Linnaeus, 1758)	LeisFerr	z	F	4	6	0	9	0	1	1	2	0	0	23	1.63%
*Leistus rufomarginatus* (Duftschmid, 1812)	LeisRufm	z	F	0	3	0	0	0	0	0	0	0	0	3	0.21%
*Licinus depressus* (Paykull, 1790)	LicnDepr	z	N	0	1	1	0	0	0	0	0	0	0	2	0.14%
*Nebria brevicollis* (Fabricius, 1792)	NebrBrev	z	F	0	6	2	0	1	0	0	0	0	0	9	0.64%
*Ophonus ardosiacus* (Lutshnik, 1922)	OphnArds	ph	F	0	1	0	0	0	0	0	0	0	0	1	0.07%
*Ophonus azureus* (Fabricius, 1775)	OphnAzur	ph	F	24	0	16	17	1	0	50	2	1	1	112	7.92%
*Ophonus sabulicola* (Panzer, 1796)	OphnSabl	ph	F	0	1	0	0	0	0	0	0	2	0	3	0.21%
*Philorhizus crucifer* (Lucas, 1846)	PhilCruc	z	F	0	0	0	0	3	1	0	3	0	0	7	0.49%
*Platynus assimilis* (Paykull, 1790)	PlayAssm	z	F	0	0	0	0	1	0	0	0	0	0	1	0.07%
*Poecilus cupreus* (Linnaeus, 1758)	PoecCupr	z	F	0	2	2	1	0	1	2	1	0	0	9	0.64%
*Pterostichus niger* (Schaller, 1783)	PterNigr	z	F	1	0	0	0	0	0	0	0	0	0	1	0.07%
*Pterostichus ovoideus* (Sturm, 1824)	PterOvoi	z	B	1	0	0	6	0	0	0	0	0	0	7	0.49%
*Pterostichus vernalis* (Panzer, 1796)	PterVern	z	F	1	0	3	2	0	0	0	0	0	0	6	0.42%
*Stenolophus discophorus* (Fischer von Waldheim, 1823)	StenDisc	om	F	0	0	0	1	0	0	0	0	0	0	1	0.07%
*Stomis pumicatus* (Panzer, 1796)	StomPumc	z	N	1	1	1	0	0	0	0	0	0	0	3	0.21%
*Syntomus pallipes* (Dejean, 1825)	SyntPall	ph	B	21	9	19	0	3	5	8	0	5	3	73	5.16%
*Tachys bistriatus* (Duftschmid, 1812)	TachBist	om	F	1	0	0	0	0	1	0	0	0	0	2	0.14%
*Tachys fulvicollis* (Dejean, 1831)	TachFulv	om	F	0	1	0	0	0	0	0	0	0	0	1	0.07%
*Trechus quadristriatus* (Schrank, 1781)	TrecQuad	z	F	13	12	15	6	2	2	15	6	12	10	93	6.57%
∑ Carabidae				427	204	204	90	24	53	243	46	80	44	1416	100%
Opiliones															
*Leiobunum rotundum* (Latreille, 1798)	LeioRotn	om	-	0	0	1	0	0	0	0	0	0	0	1	0.07%
*Nelima sempronii* Szalay, 1951	NelmSemp	om	-	1	15	0	1	12	7	0	0	0	1	37	2.62%
*Opilio saxatilis* Koch, 1839	OpilSaxt	om	-	1	62	8	85	164	653	11	136	2	104	1226	86.95%
*Phalangium opilio* Linnaeus, 1758	PhalOpil	om	-	1	32	0	0	7	3	0	0	0	1	44	3.12%
*Trogulus* agg. *tricarinatus* (Linnaeus, 1767)	TrogTric	om	-	0	2	1	5	17	11	0	0	0	0	36	2.55%
*Zachaeus crista* (Brullé, 1832)	ZachCris	om	-	2	1	0	0	35	27	0	0	0	0	65	4.61%
∑ Opiliones			-	5	112	10	91	235	701	12	136	2	106	1409	100%
∑ individuals				432	316	214	181	259	754	255	182	82	150	2825	

### Spatial dispersion

Through redundancy analysis (RDA, SD = 2.2 on the first coordination axis), we investigated the relationships between species and environmental variables (renewal/seeding, isolation, and mulching in 2022/2023). Spatial analysis explained 15.8% of the variability on the first axis and 28.71% on the second cumulative axis. After accounting for the influence of environmental variables, the variability on the first ordination axis increased to 39.44% and on the second cumulative axis to 71.7%. A statistically significant effect was observed for the variable renewal/seeding (*p* = 0.042). For the other environmental variables, no significant effects were confirmed: isolation (*p* = 0.474), mulching 2022 (*p* = 0.438), and mulching 2023 (*p* = 0.724).

**Fig. 2:**
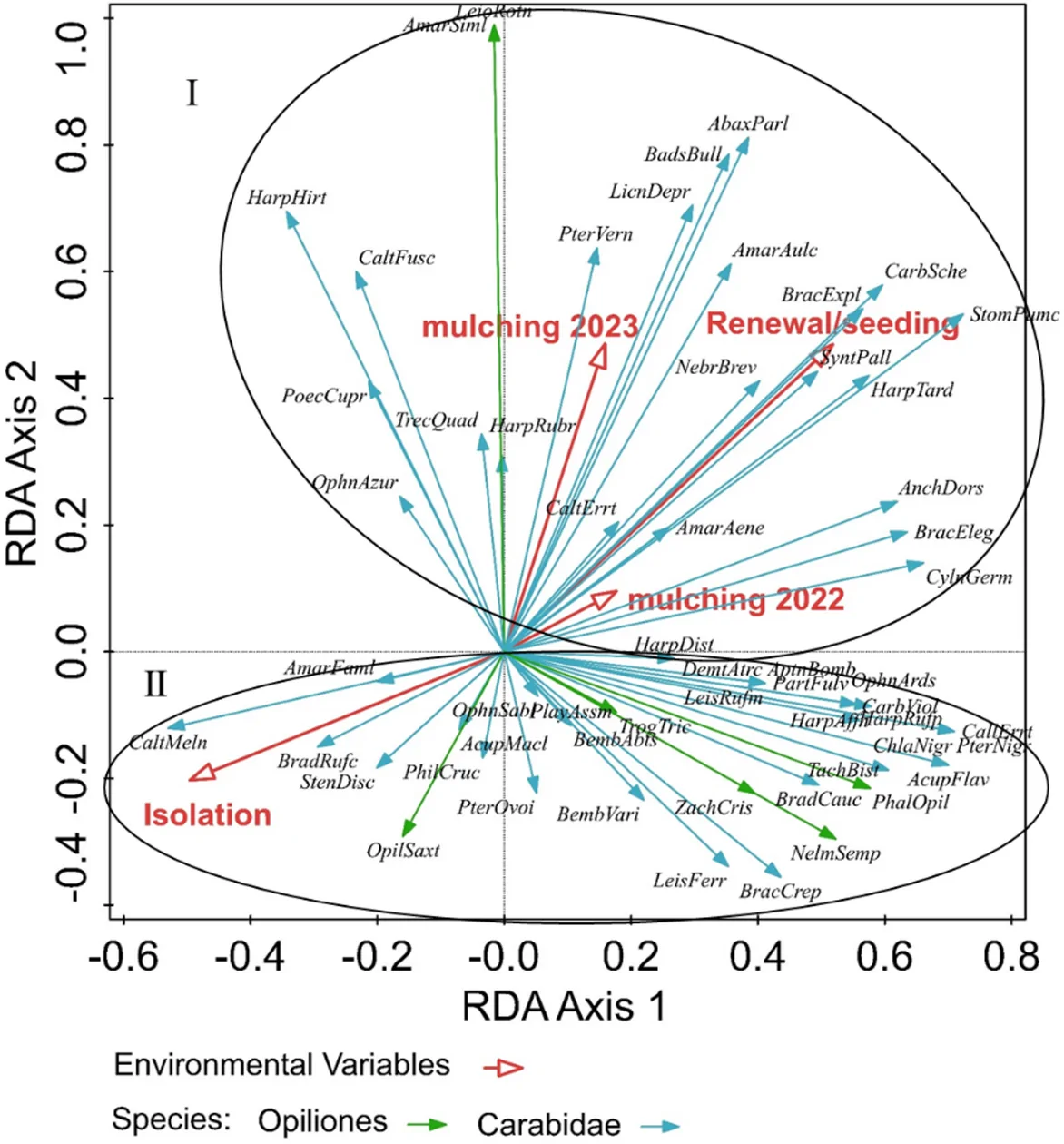
RDA analysis of species’ relationships with different types of management in the monitored habitats. The species were grouped into two main groups. The second group II consists of species that are typical of isolated habitats; the first group I consists of species that are more responsive to other factors, such as mulching or renewal/seeding itself. The abbreviations used for the taxa are explained in Table 2.

In the ordination graph, the species were organised into two clusters. In the first cluster (I), there are species that were influenced by the factor isolation. In the second cluster (II), there are species that were influenced by the remaining factors (Fig. 2).

Through redundancy analysis (RDA, SD = 2.2 on the first ordination axis), we investigated the relationships between species and environmental variables (distance from agricultural land (m), distance from forest fragment (m), and distance from the nearest road (m)). Spatial analysis explained 17.49% of the variability on the first axis and 25.4% on the second cumulative axis. After accounting for the influence of environmental variables, the variability on the first ordination axis increased to 57.81% and on the second cumulative axis to 83.94%. A statistically significant effect was observed for the variable distance from the nearest road (*p* = 0.0212). For the other environmental variables, no significant effects were confirmed: distance from agricultural land (*p* = 0.7324) and distance from forest fragment (*p* = 0.302).

**Fig. 3:**
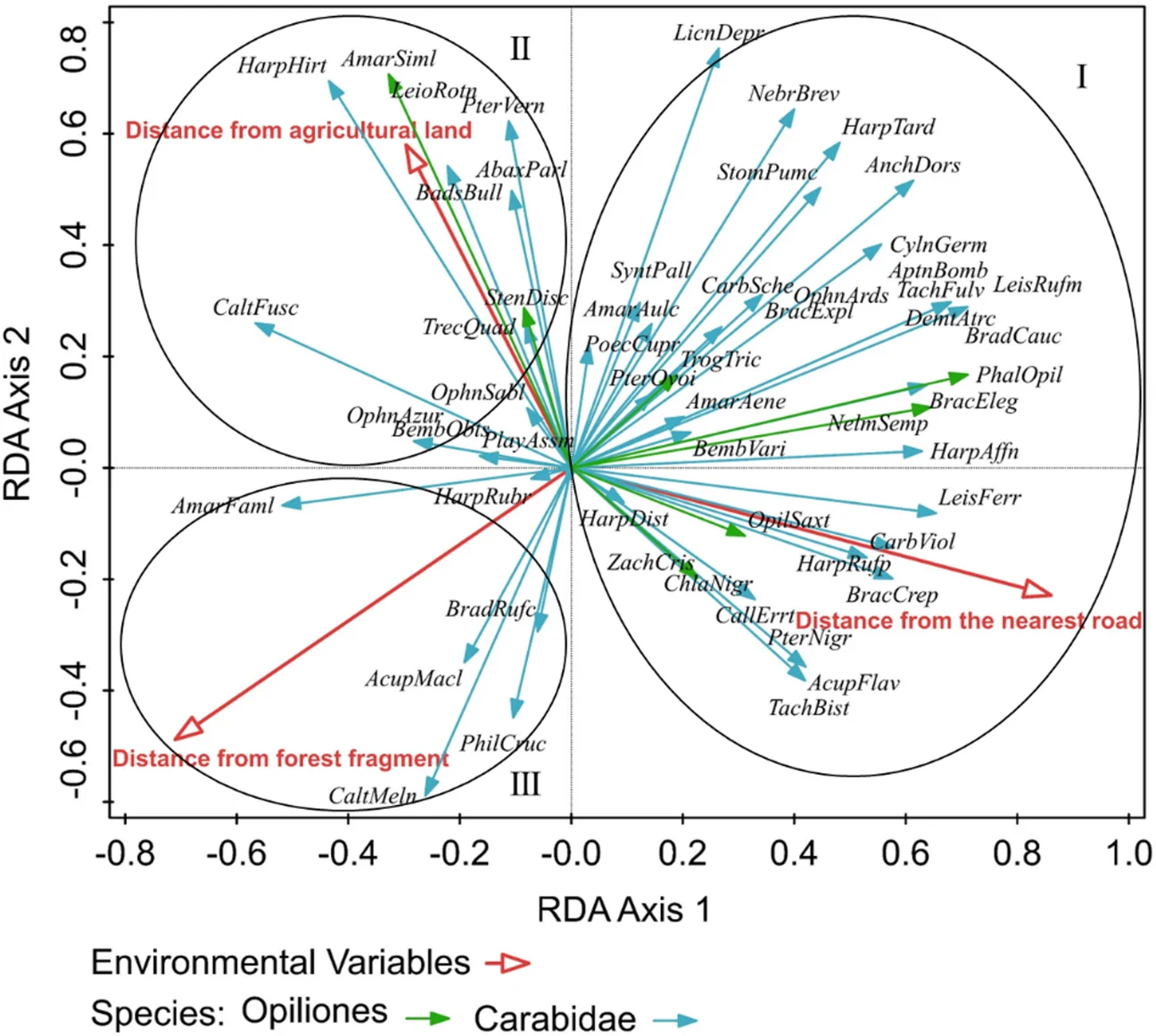
RDA analysis of species’ relationships to distance from surrounding elements of the monitored landscape. Group I consists of species that are more likely to occur further from the road. Species in another group II are more responsive to distance from agricultural land, and the third group III includes species that prefer proximity to forest fragments.

In the ordination graph, the species were organised into three clusters. In the first cluster (I), species were influenced by the factor distance from the nearest road. In the second cluster (II), there are species that were influenced by the distance from agricultural land. The third cluster (III) consists of species that preferred distances from forest fragments (Fig. 3).

Through redundancy analysis (RDA, SD = 2.2 on the first ordination axis), we investigated the relationships between species and environmental variables (E_1_ [spec.], E_2_ [spec.], E_1_ [%], E_2_ [%]). Spatial analysis explained 37.91% of the variability on the first axis and 54.04% on the second cumulative axis. After accounting for the influence of environmental variables, the variability on the first ordination axis increased to 45.39% and on the second cumulative axis to 64.69%. A statistically significant effect was confirmed for the variables E_1_ [%] 2023 (*p* = 0.194), E_1_ [%] 2022 (*p* = 0.0394), E_2_ [spec.] 2022 (*p* = 0.0418), and E_2_ [spec.] 2023 (*p* = 0.0382). For the other environmental variables, no significant effect was confirmed: E_1_ [spec.] 2023 (*p* = 0.434), E_2_ [spec.] 2022 (*p* = 0.906), E_2_ [%] 2023 (*p* = 0.578), and E_2_ [%] 2022 (*p* = 0.586).

**Fig. 4:**
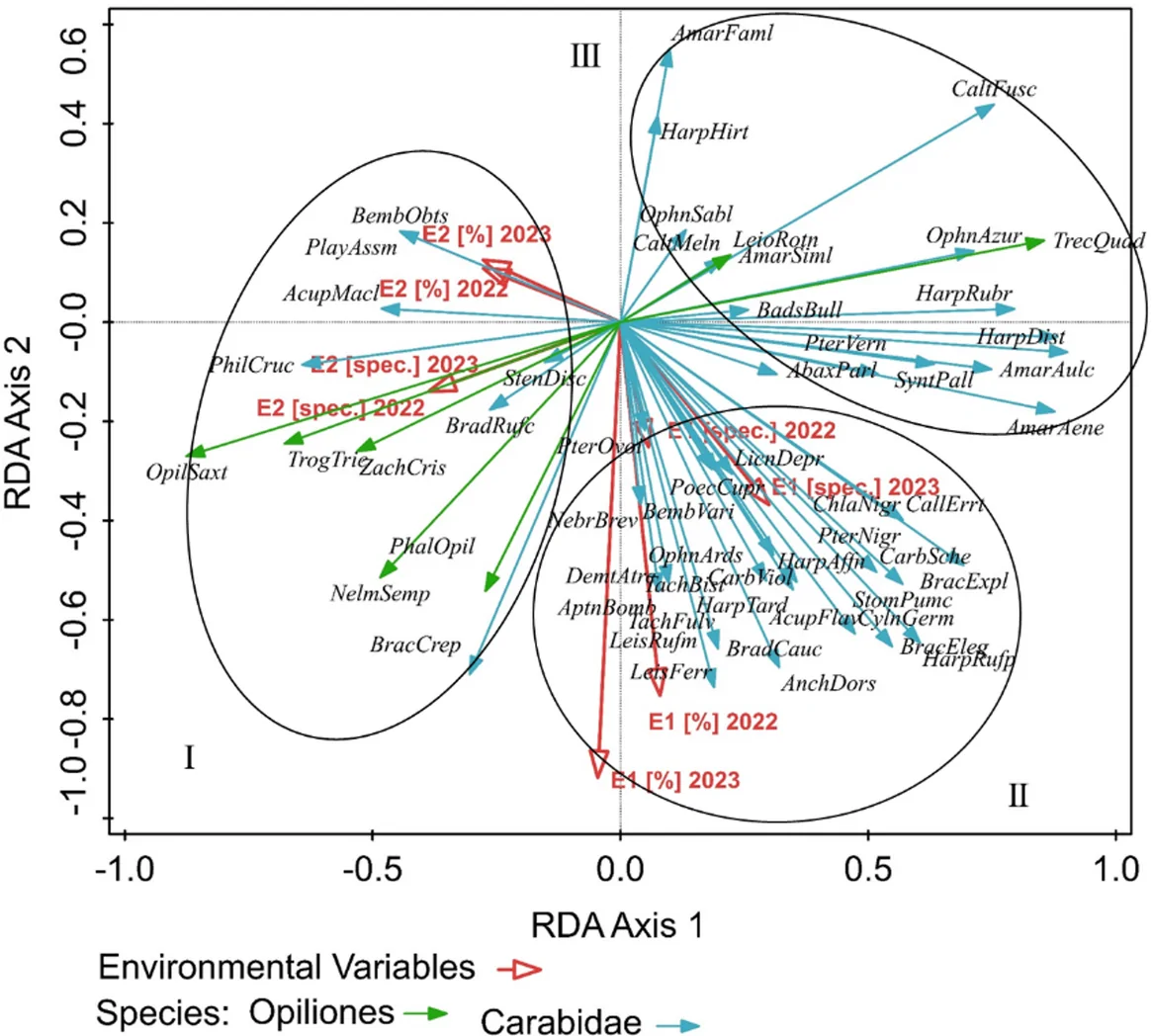
RDA analysis of species’ relationships to the structure of vegetation (coverage and species richness of the herbaceous layer and shrubs). The first group I consists of species that are most closely associated with a well-developed shrub layer, while the next group II consists of species that respond to the properties of the herbaceous layer. Group III is represented by species that do not show any significant connection to the observed vegetation characteristics.

In the ordination graph, the species were organised into three clusters. In the first cluster (I), there are species that were influenced by the factors E_2_ [%] 2023, E2 [%] 2022, E_2_ [spec.] 2022, and E_2_ [spec.] 2023. In the second cluster (II), there are species that were influenced by the remaining factors. The third cluster (III) consists of species with no preference or association with the factors (Fig. 4).

### Predictive models

**Fig. 5:**
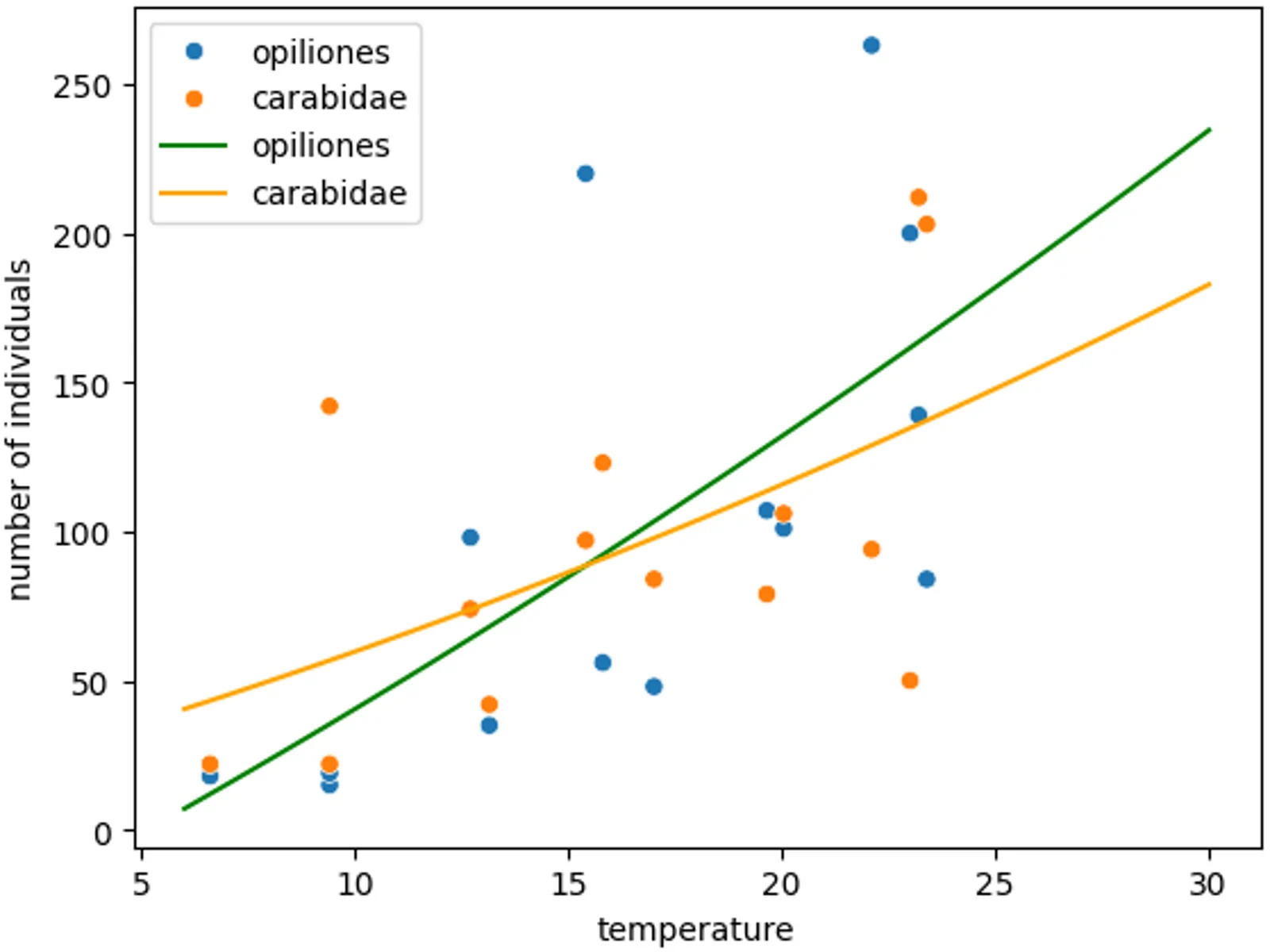
Multivariate linear regression modelling the abundance of Carabidae and Opiliones as a function of temperature. The number of individuals in both groups increased with increasing temperature, although each group reacted slightly differently.

Based on multivariate linear regression (multiple regression), we analysed the number of individuals from the Carabidae and Opiliones taxa in relation to temperature and their prediction at a temperature of 30 °C. Our model explained 58.47% of the variability (R² = 0.5847), indicating a moderately strong relationship (moderate dependency) between the variables examined. The model predicted the values of individuals at 30 °C, where there was a gradual linear increase in the number of individuals for both taxa examined. The analysis suggests that at 30 °C, there would be a greater increase in individuals of Opiliones than in Carabidae (Fig. 5).

**Fig. 6:**
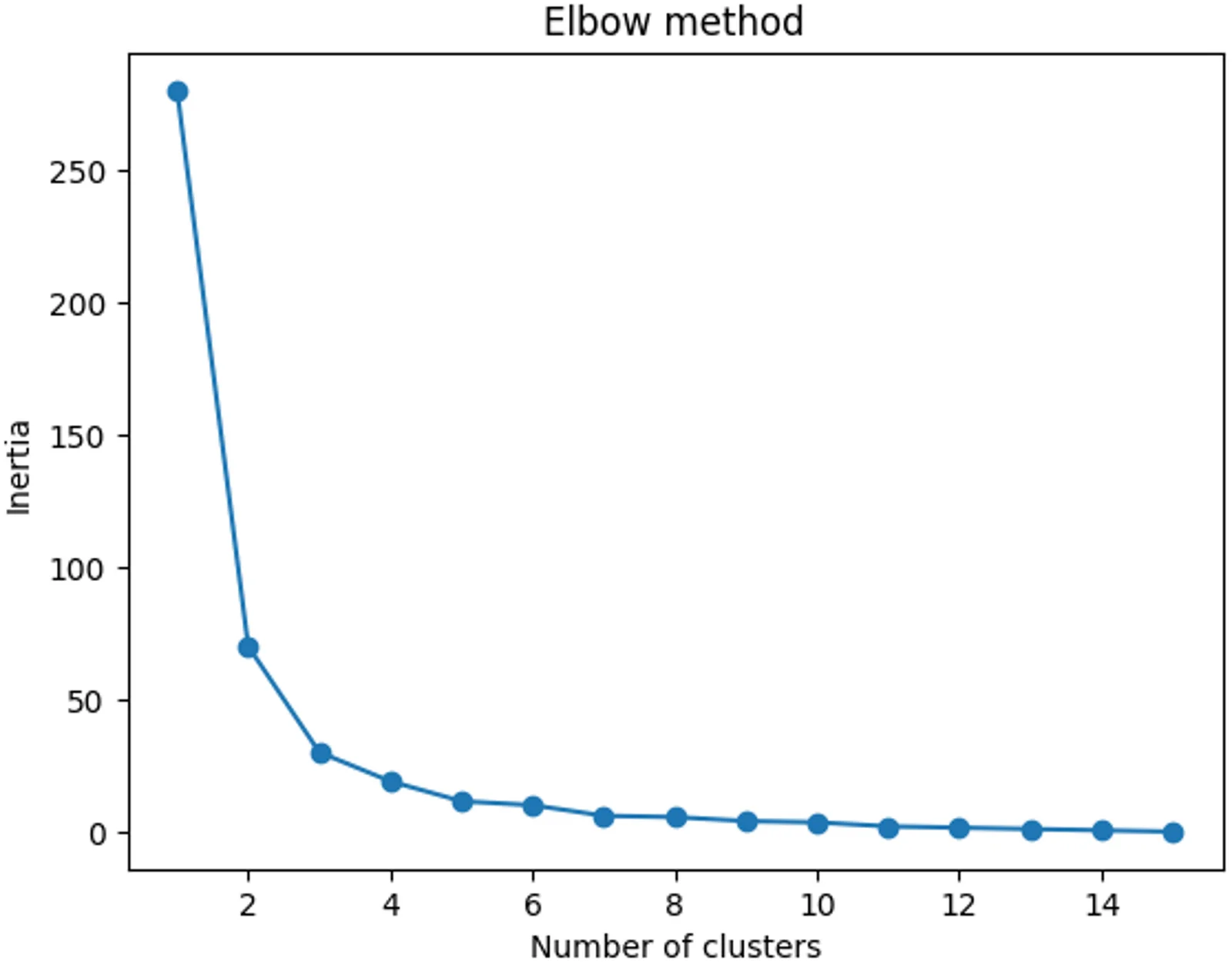
Elbow Method for determining the number of clusters (Carabidae).

Using the K-means algorithm, which is part of machine learning (a subfield of AI), we predicted the number of clusters in the time series, through which we could assess the development of Carabidae species at the study sites. First, we determined the appropriate number of clusters for our data analysis using the Elbow Method. This method calculates clusters by summing squared errors (SSE) to find the point where increasing the number of clusters results in diminishing returns in terms of reducing the SSE. The results showed that the optimal number of clusters was three (Fig. 6).

**Fig. 7:**
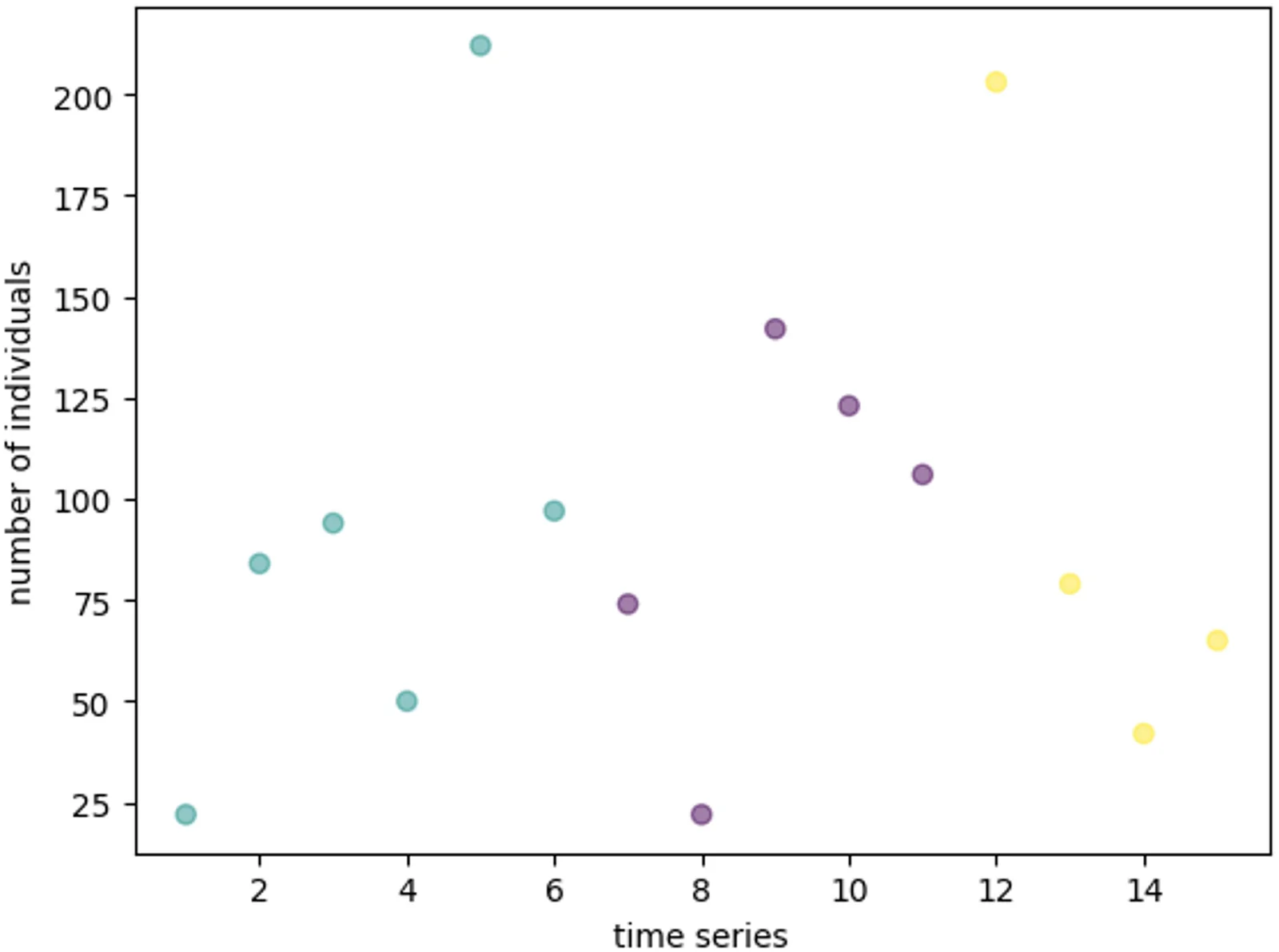
Cluster prediction using the K-means algorithm. Explanations: Numbers 1 to 6 = Year 2022 and months 4–8; Numbers 7 to 11 = Year 2022 and months 9–11, Year 2023 and months 4, 6; Numbers 12 to 16 = Year 2023 and months 7–10, Year 2024 and month 1.

Using the K-means algorithm, we predicted three clusters within the time series. Based on these, we can conclude that there were gradual positive changes in the abundance of individuals from the Carabidae family at the study sites, as their numbers increased. Three periods of increased abundance were identified: the first period is between intervals 1–6, which corresponds to the year 2022 and the months of April to September. The next cluster is between intervals 7–11, which corresponds to the year 2022 and the months of October to November, and the year 2023 and the months of April to June. The final cluster is between intervals 12–15, corresponding to the year 2023 and the months of July to October, and the year 2024 and the month of January (Fig. 7).

**Fig. 8:**
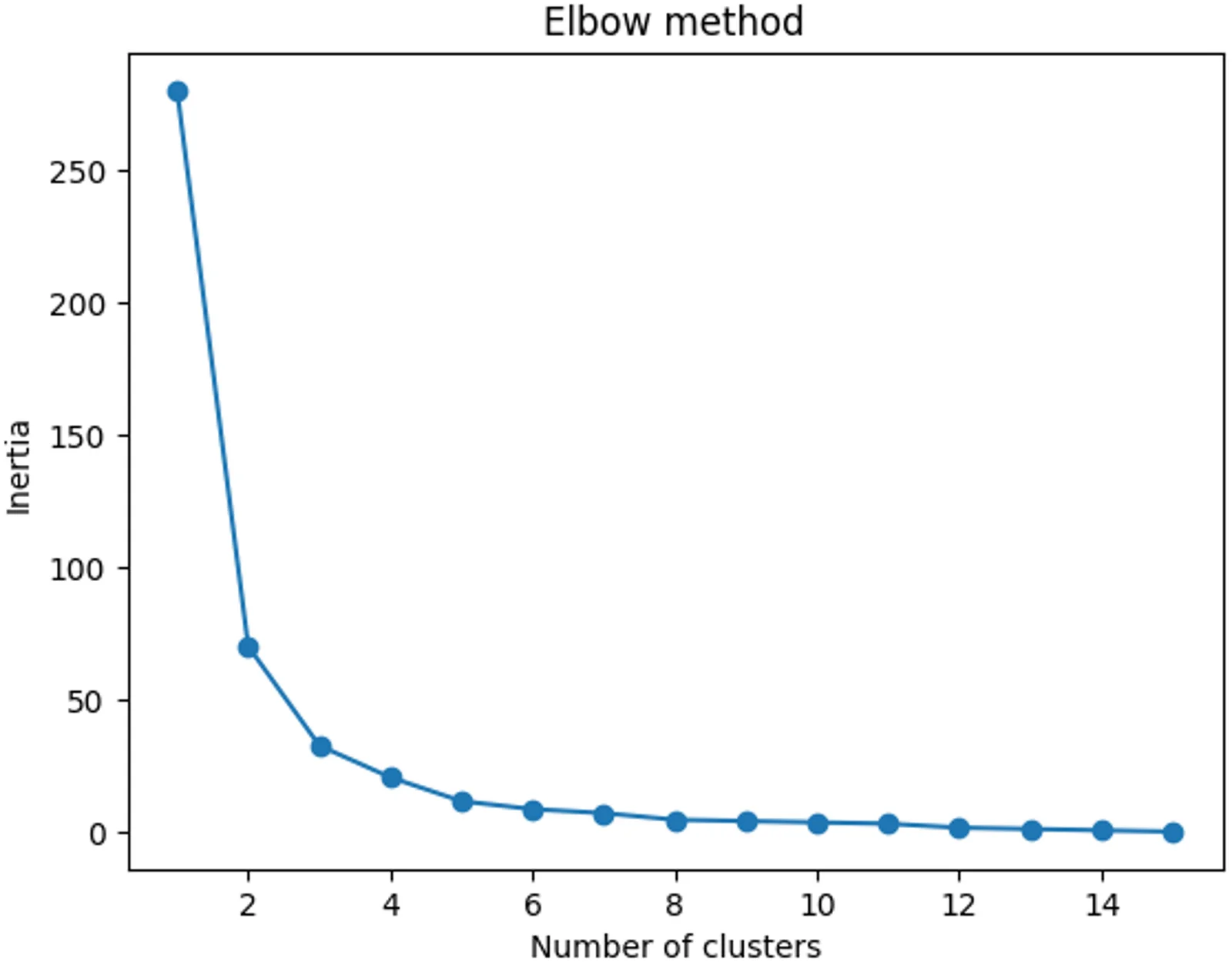
Elbow Method for determining the number of clusters (Opiliones).

Using the K-means algorithm, which is part of machine learning (a subfield of AI), we predicted the number of clusters in the time series, through which we can assess the development of Opiliones on the study sites. First, we determined the appropriate number of clusters for our data analysis using the elbow method. This method calculates clusters by summing squared errors (SSE), to find the point where increasing the number of clusters results in diminishing returns in terms of reducing SSE. The results show that the optimal number of clusters is three (Fig. 8).

**Fig. 9:**
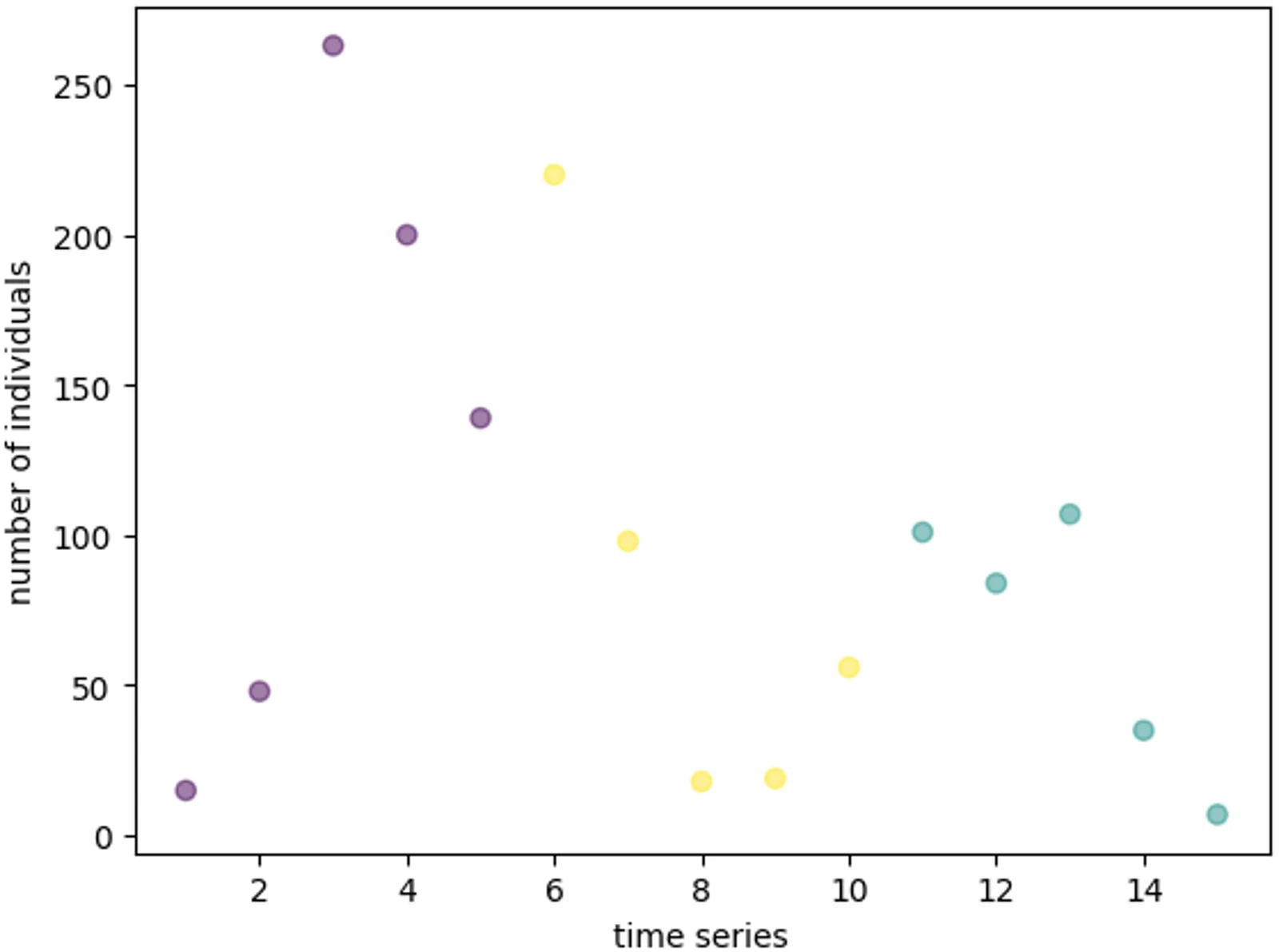
Cluster prediction using the K-means algorithm. Explanations: Numbers 1–5 = Year 2022 and months 4–8; Numbers 6–10 = Year 2022 and months 9–11, Year 2023 and months 4, 5; Numbers 11–15 = Year 2023 and months 6–10, Year 2024 and month 1.

Using the K-means algorithm, we predicted three clusters within the time series. Based on these, we can conclude that there were gradual negative changes in the abundance of individuals of the order Opiliones at the study sites, as their numbers decreased. Three periods of decreased abundance were identified: the first period is between intervals 1–5, corresponding to the year 2022 and the months of April to August. The subsequent cluster is between intervals 6–10, corresponding to the year 2022 and the months of September to November, and the year 2023 and the months of April and May. The final cluster is between intervals 11–15, corresponding to the year 2023 and the months of June to October, and the year 2024 and the month of January (Fig. 9).

## Discussion

Our two-year study at the Nitra-Selenec expressway junction reveals complex, trait-specific responses of epigeic arthropods to roadside habitat management. The contrasting patterns observed between Carabidae (50 species, 1,416 individuals) and Opiliones (6 species, 1,409 individuals) reflect fundamental differences in their trophic ecology and dispersal capacity, which interact with management practices, vegetation structure, and road proximity to shape community assembly.

### Trophic Strategies and Resource Availability

The dominance of *Opilio saxatilis* (86.95% of all harvestmen) across nearly all sites, with particularly high abundances at S6 (653 individuals), S5 (164 individuals) and S8 (136 individuals), provides compelling evidence supporting the “road subsidy” hypothesis. Recent research conducted at the same expressway junction^43^demonstrates that high-speed roads generate substantial arthropod mortality through vehicle collisions, with dead insects occurring at significantly elevated densities in proximity to road sections. Hymenoptera accounted for 75% of road-killed insects, followed by Diptera (17.5%) and Coleoptera (4.4%). For generalist scavengers like harvestmen, this constitutes a predictable and abundant food resource. Mediation analysis by Litavský et al.^43^, showed that high-speed roads influence harvestmen abundance indirectly via increased availability of insect carrion. This pattern explains the exceptional abundance of *O. saxatilis* at S6 (situated only 3.1 m from the expressway). S8 (2.4 m from the expressway) and S5 (23.2 m from the road, where 60–70% shrub cover provides structural refuge). The species’ omnivorous feeding strategy allows it to exploit both live prey and carrion, granting a distinct advantage in roadside environments where insect mortality is elevated.

In contrast, ground beetles (Carabidae) exhibited the highest abundance at sites with a lower intensity of management and a greater distance from the edges of the road. Study sites located in the central parts of the monitored area, such as: S1 (427 individuals), S7 (243 individuals), S2 and S3 (204 individuals each), supported the largest populations of ground beetles. The eudominant species, *Harpalus distinguendus* (16.82%), *Harpalus rufipes* (13.99%), and *Harpalus rubripes* (9.19%), are omnivorous, capable of exploiting both animal and plant resources^53^. This trophic flexibility may buffer these species against resource fluctuations in disturbed roadside environments. However, the predominance of zoophagous species such as *Calathus erratus* (5.86%), *Calathus fuscipes* (6.22%), and *Trechus quadristriatus* (6.57%) at sites with complex vegetation structure (S1, S2, S3, S7) indicates that predation on live invertebrates remains the primary feeding mode for many carabids, and that these species tend to avoid the most heavily disturbed, structurally simplified road-edge habitats.

### Dispersal ability and habitat connectivity

The contrasting dispersal capacities of Carabidae (mostly flying) and Opiliones (flightless) emerge as a critical filter shaping community responses to management and isolation.

Among carabids, flying species dominated the assemblage (Table 2). Of the 50 species recorded, 41 are capable of flight (F) and 6 are brachypterous, but occasionally able to fly (B). Only three species, *Abax parallelepipedus*, *Aptinus bombarda*, and *Stomis pumicatus*, are permanently flightless (N). This high proportion of macropterous species facilitates colonisation of isolated habitat patches, which explains why the “Isolation” variable was not statistically significant (*p* = 0.474) in our RDA analysis. Even completely isolated vegetation islands (S7–S10) were readily colonised by flying carabids, as demonstrated by the 243 individuals of S7 representing diverse flight-capable species including *Harpalus distinguendus* (38), *Ophonus azureus* (50), and *Calathus erratus*(24). Niemelä and Spence^58^, demonstrated that newly established carabid populations are characterised by high proportions of macropterous (long-winged) individuals, with dispersal from source populations occurring primarily through flight. Our findings align with this pattern: Rapid colonisation of renewal/seeding sites (S1, S4, S7) by flying species indicates that management interventions that create open habitats are quickly exploited by mobile carabids, even when these sites are isolated by road infrastructure.

In contrast, harvestmen are entirely flightless and less mobile^35,43^. Therefore, their distribution should reflect local habitat quality and landscape connectivity more strongly than that of carabids. The highest abundances of harvestmen occurred at S6 (701), S5 (235), S2 (112), and S8 (136), all sites with either proximity to the road (providing food subsidy) or structural complexity (providing refuge). S6, despite its extremely close proximity to the expressway (3.1 m), supported the largest harvestmen population, suggesting that the benefits of carrion subsidy outweigh the negative effect of disturbance for these scavengers. However, the complete absence of harvestmen from some isolated, intensively managed sites (e.g., S9 with only 2 individuals) indicates that flightless arthropods exhibit substantially greater sensitivity to habitat fragmentation and intensive management. Quiles and Barrientos^59^, emphasise that roads disrupt multiple interspecific interactions and that, for less mobile taxa, such as harvestmen, both resource availability and habitat connectivity must be considered in conservation planning.

### Management intensity and vegetation structure

The significant negative effect of “renewal/seeding” (*p* = 0.042) on community composition reflects the homogenising impact of commercial seed mixtures dominated by grasses (90%). S1, S4, and S7—all renewal/seeding sites—showed distinct carabid assemblages dominated by agrobiont, open-habitat species such as *Harpalus distinguendus*, *H. rufipes*, and *Ophonus azureus*^45,46^. These species are typical of arable landscapes and early successional stages, but the assemblages lack the forest-associated and specialist species found in passively managed sites.

S2 and S5, both passively managed with diverse shrub layers (*Crataegus* sp., *Ligustrum* sp., *Prunus spinosa*), supported more balanced communities. At the site S2 we recorded 204 carabids in 27 species, including forest-associated *Carabus violaceus* and *Abax parallelepipedus*, alongside open-habitat species. The S5 harvestmen population (235 individuals) was dominated by *O. saxatilis*, but also contained *Zachaeus crista* (35) and *Nelima sempronii* (12), demonstrating that structurally complex vegetation provides microhabitat heterogeneity supporting multiple harvestmen species.

The vegetation structure emerged as the strongest positive driver of community assembly. Herb layer cover (E_1_ [%] 2022, *p* = 0.0394; 2023, (*p* = 0.194)) and shrub layer species richness (E_2_ [spec.] 2022, *p* = 0.0418; 2023, *p* = 0.0382) were all statistically significant. S7, despite being a renewal/seeding site, recorded the highest plant species richness in the herb layer (37 species in 2022, 36 in 2023) and supported 243 carabids and 12 harvestmen, demonstrating that even within active management regimes, high botanical diversity can mitigate some negative impacts. Conversely, S10 (intensively mulched, isolated, with lowest plant species richness (16 species) and dominated by *Elytrigia repens*) supported only 44 carabids and 106 harvestmen (almost exclusively *O. saxatilis*). This represents a severely depauperate community.

Haas-Renninger^60^, demonstrated that conventional mowing has highly detrimental effects on arthropod fauna, but these effects can be mitigated by arthropod-friendly mowing techniques and reduced mowing frequency. Our results strongly support this: extensively mulched sites (S1, S4, S6, S7, S8) generally supported higher diversity than intensively mulched sites (S3, S9, S10). Quiles and Barrientos^59^, recommend combining different mowing regimes to increase the complexity of the habitat corridor, allowing the road verges to function as habitat for more species.

Effective management measures, such as reduced frequency and timing of mowing, selective planting of vegetation, and the creation of microhabitats, significantly improve invertebrate diversity in roadside environments^61–63^.). For instance, less intensive mowing allows flowering plants to establish, supporting pollinators like bees and butterflies^64^; Noordijk et al.^8^,. These findings fully align with our results.

### Future habitat development and agroforestry analogies

Beyond current management practices, the trajectory of habitat development at our study sites warrants consideration. The successional processes along roadside verges represent a key mechanism to improve habitat quality and connectivity for epigeic arthropods. As vegetation matures and structural complexity increases, the diversity of microhabitats expands, providing refugia, foraging grounds, and overwintering sites for a wider range of species^65^. Trees have been planted in the central parts of the monitored area (sites S1, S3, S7 and S9), including species such as *Acer campestre*, *A. platanoides*, *A. pseudoplatanus*, *Betula pendula*, *Fraxinus excelsior*, *Padus racemosa*, *Sorbus aucuparia*, and *Tilia platyphyllos*. As these trees mature and reach the height of the tree layer, the habitat will acquire structural characteristics similar to traditional European agroforestry systems^66,67^. In terms of vegetation structure, these plantings will increasingly resemble traditional orchards, which combine trees with grass-herb undergrowth, effectively bridging the conditions of meadow and forest environments^68^. This structural succession will likely influence epigeic arthropod communities further, potentially increasing habitat suitability for forest-associated carabids (e.g., *Abax parallelepipedus*, *Carabus violaceus*) and providing additional microhabitats for harvestmen. Importantly, mature tree avenues along roads can function as effective migration corridors, particularly for flightless forest-dwelling carabid species. Research on the A-2 motorway in the Wielkopolska region of Poland demonstrated that road verges lined with approximately 80–100-year-old tree avenues in an open agricultural landscape (where the landscape matrix consisted of arable fields with isolated forest patches) likely functioned as migration corridors for flightless epigeic carabid species living in forests^69^. Individuals of these Carabidae species found along asphalt roads bordered by tree-lined avenues appeared to be attempting to move between adjacent, isolated forest patches^69^. This finding supports our prediction that the maturing tree plantings at our study sites (S1, S3, S7, S9) may increasingly function as ecological corridors over time, enhancing connectivity in anthropogenically fragmented landscapes. Furthermore, the formation of such corridors would be particularly beneficial for less mobile species, including harvestmen and flightless carabids, which are otherwise vulnerable to habitat fragmentation^30,36^. The existing knowledge on the management of traditional orchards^70^ and similar agroforestry systems thus offers a valuable foundation for developing appropriate long-term management strategies for these human-created plantings near road infrastructure. Adaptive management approaches that maintain herb-layer diversity while permitting the development of the tree layer will be essential to maximise biodiversity gains as these novel habitats mature.

### Road proximity effects

The significant effect of “distance from the nearest road” (*p* = 0.0212) on community composition reflects the dual nature of roads as stressors and resource subsidies^20,43,59^. S6 (3.1 m from the road) and S8 (2.4 m from the road) illustrate this paradox: both sites supported large harvestman populations (701 and 136 individuals, respectively) due to food subsidies, but relatively low carabid diversity (S6: 53 individuals, 14 species; S8: 46 individuals, 14 species). Carabids, particularly predators, appear to avoid the immediate road edge, consistent with findings that carabid abundance decreases near roads due to avoidance of disturbance^43,71^.

S3, located farthest from the nearest road (60.2 m) and closest to a forest fragment (50.6 m), supported 204 carabids dominated by forest-associated and predatory species including *Calathus fuscipes* (57), *C. erratus* (6), and *Carabus violaceus*(1). This suggests that the distance from the disturbance of the road, combined with the proximity to the source populations in adjacent habitats, allows less mobile or disturbance-sensitive species to persist. Rebrina^71^, found that the proximity of a motorway affects ground beetle assemblages primarily through changes in vegetation structure, combined with traffic noise effects. Our data support this: the sites closest to the road (S4: 2.8 m, S8: 2.4 m, S10: 2.0 m) had a simplified vegetation structure (low E_2_ cover/species richness) and correspondingly simplified arthropod communities dominated by a few disturbance-tolerant species.

This suggests that distance from the disturbance of the road, combined with the proximity to source populations in adjacent habitats, enables the persistence of less mobile and disturbance-sensitive species. Our data support this: sites nearest to the roadway exhibited markedly simplified vegetation structure and correspondingly reduced arthropod diversity, with communities dominated by a small number of disturbance-tolerant taxa.

### Temporal trends

The K-means cluster analysis revealing increasing carabid abundance but declining harvestmen populations over the study period warrants careful interpretation. The gradual increase in carabids may reflect community stabilisation following 2021 renewal/seeding interventions, with mobile species colonising and establishing populations. The three identified clusters (April–September 2022; October–November 2022 + April–June 2023; July–October 2023 + January 2024) suggest ongoing community dynamics rather than simple linear trends.

The trend of declining harvestmen is more concerning and may indicate that even generalist scavengers face long-term challenges in roadside environments. Possible explanations include: (1) cumulative effects of repeated mulching that reduce the litter layer and soil moisture essential for harvestmen^35,60^; (2) pesticide drift from adjacent agricultural land (notably S1, which is closest to agricultural land); (3) road mortality of harvestmen themselves, which may offset the benefit of food subsidies over time; or (4) climatic factors during the study period. Longer-term monitoring is essential to determine whether this decline represents a temporary fluctuation or a persistent negative trend.

### Implications for management and conservation

Our findings have direct implications for roadside management at the Nitra-Selenec junction and similar expressway verges across Central Europe. First, the clear negative effect of renewal/seeding with commercial grass-herb mixtures indicates that where biodiversity conservation is the primary management objective, passive management or the targeted use of native seed mixtures should be prioritised over commercial revegetation. Second, maintaining and improving the structural complexity of the vegetation, particularly the diversity of the shrub layer and the cover of the herb layer, is the most effective strategy to support the diverse communities of epigeic arthropods. Third, a mosaic management approach that combines: (a) safety zones with intensive mowing immediately adjacent to the road; (b) extensive mowing (1–2 times a year at ≥ 10 cm height) in intermediate zones; and (c) passive management with the development and maintenance of the shrub layer in the interior areas of the vegetation would maximise habitat heterogeneity and support flying carabids, as well as less mobile harvestmen.

The contrasting responses of these two indicator groups underscore the importance of multi-taxa approaches in environmental monitoring and assessment. Management decisions based solely on carabids might overlook negative impacts on harvestmen, and vice versa. As Quiles and Barrientos^59^ emphasise, roads disrupt multiple interspecific interactions, and effective conservation requires a detailed understanding of these complex dynamics.

## Conclusion

This two-year monitoring study at the Nitra-Selenec expressway junction demonstrates that roadside habitat management profoundly influences epigeic arthropod communities, with effects likely mediated by vegetation structure, proximity to the road and species traits. Active revegetation with commercial grass-herb mixtures significantly negatively affected community composition for both Carabidae and Opiliones, creating homogeneous habitats dominated by generalist species. In contrast, the cover of the herb layer and the species richness of the shrub layer were the most significant positive drivers of arthropod diversity, underscoring that the structural complexity of the vegetation provides essential microhabitats and shelter from disturbance. Road proximity exerted a dual influence: distance from the road significantly shaped species distributions, with scavenging harvestmen benefiting from food subsidies, while predatory carabids avoided immediate road edges. Temporal analysis revealed divergent population trends—increasing carabid abundance but declining harvestmen—highlighting the necessity of long-term, multi-taxa monitoring for accurate environmental assessment. As trees planted at our study sites mature, these habitats will increasingly resemble traditional European agroforestry systems, offering opportunities for adaptive management informed by orchard conservation knowledge.

Where biodiversity conservation is the objective, we recommend that road authorities adopt: (1) passive management or regionally appropriate native seed mixtures instead of commercial revegetation; (2) reduced frequency of mowing (1 to 2 times a year at ≥ 10 cm height) to preserve herb layer diversity; (3) retention and enhancement of shrub layer vegetation to increase structural complexity; (4) mosaic management combining intensive mowing only in safety-critical zones with extensive management elsewhere; and (5) establishment of buffer zones at least 3 m from road edges to reduce disturbance impacts on predatory arthropods. These evidence-based measures will transform expressway verges from ecological traps into functional refuges for the biodiversity of invertebrates.

## Data Availability

All data produced from this study are provided in this manuscript.
